# Healthy longevity-associated protein improves cardiac function in murine models of cardiomyopathy with preserved ejection fraction

**DOI:** 10.1186/s12933-024-02487-6

**Published:** 2024-11-05

**Authors:** Valeria Vincenza Alvino, Sadie Slater, Yan Qiu, Monica Cattaneo, Khaled Abdelsattar Kassem Mohammed, Seamus Gate, Vealmurugan Sekar, Annibale Alessandro Puca, Paolo Madeddu

**Affiliations:** 1 Bristol Medical School, Translational Health Sciences, University of Bristol, Upper Maudlin St, Bristol, BS2 8HW UK; 2 Cardiovascular Department, IRCCS MultiMedica, Via G. Fantoli, 16/15, 20138 Milan, Italy; 3 Department of Cardiothoracic Surgery, Faculty of Medicine, Assiut University, Assiut, Egypt; 4 Department of Medicine, Surgery and Dentistry “Scuola Medica Salernitana”, University of Salerno, Via Salvatore Allende, 84081 Baronissi Salerno, Italy

## Abstract

**Aims:**

Aging is influenced by genetic determinants and comorbidities, among which diabetes increases the risk for heart failure with preserved ejection fraction. There is no therapy to prevent heart dysfunction in aging and diabetic individuals. In previous studies, a single administration of the longevity-associated variant (LAV) of the human *BPIFB4* gene halted heart decline in older and type 2 diabetic mice. Here, we asked whether orally administered LAV-BPIFB4 protein replicates these benefits.

**Materials and Methods:**

In two controlled, randomized studies, 18-month-old male C57BL/6 J mice and 9-week-old C57BLKS/J-Leprdb/Leprdb/Dock7 + [db/db] mice of both sexes underwent baseline echocardiography. They then received a recombinant purified LAV-BPIFB4 protein (3 µg/animal, every three days) or vehicle by gavage. After 30 days, the animals underwent echocardiography, and the hearts were collected post-termination for histology.

**Results:**

All the animals completed the study except one female diabetic mouse, which was culled prematurely because tooth malocclusion caused eating problems. There was no effect of the LAV-BPIFB4 protein on body weight in the two studies or glycosuria in the diabetic study. In aging mice, LAV-BPIFB4 increased myocardial Bpifb4 expression, improving heart contractility and capillarity while reducing perivascular fibrosis and senesce. In male diabetic mice, LAV-BPIFB4 therapy improved systolic function, microvascular density, and senescence, whereas the benefit was limited to systolic function in females.

**Conclusions:**

This study shows the feasibility and efficacy of a variant protein associated with human longevity in contrasting pivotal risk factors for heart failure in animal models. The diabetic study revealed that sex influences the treatment efficacy.

**Supplementary Information:**

The online version contains supplementary material available at 10.1186/s12933-024-02487-6.

## Introduction

Heart failure with preserved ejection fraction (HFpEF) is becoming increasingly prevalent in industrialized countries, a trend influenced by an aging population and comorbidities [1–3]. In addition to older age, risk factors for HFpEF include hypertension, diabetes, dyslipidemia, and obesity. Defined as HF with an EF of 50% or higher, HFpEF affects approximately 3 million people in the US and 32 million worldwide [4, 5]. Observational multicohort studies suggest a trend toward a greater incidence in women after adjusting for risk factors [6, 7]. Patients with HFpEF are hospitalized 1.4 times per year and have an annual mortality rate of approximately 15% [2]. Female sex is independently associated with an increased risk of the clinical endpoint, which is driven mainly by the association with HF readmission [8].

The management of HFpEF is challenging because of its complex pathophysiology [9]. In older people, EF is maintained by drawing on and exhausting all reserve heart resources. In obese individuals, the heart fails to meet the circulatory and energetic demands associated with excess body weight [10]. In both conditions, low-grade inflammation plays a key pathogenic role [11]. There is no medical therapy capable of reducing cardiovascular or all-cause mortality in patients with chronic HF at the higher range of EF [12]. Sodium-glucose cotransporter type 2 (SGLT2) inhibitors and the angiotensin receptor neprilysin inhibitor (ARNi) sacubitril-valsartan, considered first-line treatments for mildly reduced EF, are mainly effective in reducing the rate of HFpEF hospitalizations [13–15]. The preventive efficacy of these pharmacological treatments for at-risk people remains undefined. [16]

Epidemiological studies have shown that some exceptional supercentenarians are seemingly protected from cardiovascular disease [17, 18]. Understanding the unusual genetic determinants behind supercentenarians’ cardiovascular fitness could help improve the prevention and precision treatment of cardiomyopathies.

We reported that homozygous carriers of a variant of the bactericidal/permeability‐increasing fold-containing family B member 4 (*BPIFB4*) gene have a greater probability for prolonged health span, manifested by physical, cardiovascular, and immune fitness, compared with age-matched peers [19–21]. These advantages could be transferred through *BPIFB4* gene therapy to animal models of cardiovascular disease. A single administration of the *LAV-BPIFB4* gene protected mice from atherosclerosis, hypertension, and myocardial and limb ischemia [20, 22]. Moreover, *LAV-BPIFB4* gene therapy has shown pleiotropic therapeutic effects in murine models of diabetic and aging cardiomyopathy, improving echocardiography indices of cardiac function, enhancing microvascular density, and reducing lipid accumulation and fibrosis in the myocardium [23, 24]. At the molecular level, the transduced LAV-BPIFB4 protein induced eNOS activation and calcium mobilization through PERK- and PKCα-dependent phosphorylation of the serine 75 [25], thereby resulting in a cascade of benefits related to ribosomal biogenesis, DNA stability, proteostasis, and the immune response [20–24, 26].

Proteins are effective biotherapeutics and have several advantages over gene therapy, especially for prolonged treatments [27, 28]. In the present study, we investigated the possibility that the LAV-BPIFB4 protein protects cardiac health in older and obese mice with type 2 diabetes. Results show that LAV-BPIFB4 therapy can benefit both conditions, indicating that this longevity-associated protein can antagonize two prevalent risk factors for HF.

## Experimental details

### BPIFB4 protein constructs

Hek-293 cells were transfected with the *LAV-BPIFB4* vector cloned in fusion with His-Tag. The recombinant protein was purified using affinity Nuvia IMAC Resin (Bio-Rad). We used the eluate of cells transfected with the null vector as a control. [23]

### In vivo studies

Two controlled, randomized studies followed the EU Directive 2010/63/EU and the principles stated in the Guide for the Care and Use of Laboratory Animals (Institute of Laboratory Animal Resources, 1996). The procedures detailed below were prepared with support from the Experimental Design Assistant, a free resource from the National Centre for Replacement, Refinement, and Reduction (3Rs) of Animals in Research 166 (https://eda.nc3rs.org.uk/), were covered by an ethical license approved by the British Home Office and the Universities of Bristol and Cardiff (# PP1377882, valid until October 25, 2025). They were deposited, before starting the experimentation, on The Animal Study Registry (Title: Transferring healthy longevity gene to improve age-related heart dysfunction, Doi 10.17590/asr.0000316, Date of registration:2023-04-26, www.animalstudyregistry.org). The principal investigator (PM) will provide access to the deposited protocol and experimental data upon request.

*Maintenance and randomization.* The mice were housed in groups of 1–6 subjects (as required by the experimental procedure) in an enriched environment within a biosecure unit under a 12-h light/dark cycle. They were fed with EURodent Diet (5LF5, LabDiet, Durham, UK) and given drinking water ad libitum. The GraphPad software (https://www.graphpad.com/quickcalcs/randomize) randomly assigned the mice to the control and treatment groups (fixed ratio = 1:1).

*Methods to minimize bias*. The LAV-BPIFB4 protein and vehicle were administered by an investigator blinded to the intervention status (VVA). Similarly, investigators (VVA, YQ, and SS) blinded to the randomization performed the echocardiography and histological analyses. The animals were exposed to the same enriched environment and received the same care and observation to avoid performance bias. An intention-to-treat approach was applied to maintain the integrity of randomization and strengthen the trial's internal validity. In line with this, participants in the two arms of the study (vehicle or LAV-BPIFB4 protein) were considered in the analysis of the echocardiography data regardless of whether they did not complete the follow-up due to premature termination.

*Treatment.* Following baseline echocardiography (Vevo 3100, VisualSonics), 18-month-old C57BL/6 J mice (Charles River, UK) were randomized to receive a recombinant purified LAV-BPIFB4 protein (100 μL of 3 µg/ animal) or an equivalent volume of vehicle by gavage every three days under isoflurane anesthesia (2–3%). Our previous study using *LAV-BPIFB4* gene therapy did not reveal any difference in the treatment effect between older male and female mice. Therefore, the present LAV-BPIFB4 protein therapy was conducted in male mice only. On the other hand, whether sex influences LAV-BPIFB4 therapy under diabetic conditions has not been previously tested [24]. Therefore, the same LAV-BPIFB4 protein or vehicle administration protocol was used for 9-week-old male and female C57BLKS/J-Leprdb/Leprdb/Dock7 + [db/db] mice. Animals were terminated at the 30-day follow-up after a final echocardiography assessment under isoflurane anesthesia by exsanguination, followed by removal of the heart for histology. In a pilot study on elderly mice, we found that a 2-week treatment was sufficient to appreciate the benefit of LAV-BPIFB4 therapy on echocardiography and histology endpoints. Nonetheless, we decided to extend the follow-up to 30 days to maximize the information on efficacy and cross-compare our findings with those of our *LAV-BPIFB4* gene therapy studies, which had the same duration [23, 24]. Previous reports have shown that repeated four-week gavages are well tolerated and do not cause metabolic effects in leptin-deficient ob/ob mice with type 2 diabetes. [29, 30]

*Endpoints.* Primary: indices of cardiac function; secondary: microvascular density, fibrosis, and senescence.

#### Determination of glucose levels

Blood glucose was determined at the end of the study via a blood glucose monitor (Accu-Check Aviva Blood Glucose Meter/Monitor, UK). Since this machine's range is up to 34 mmol/l, and blood glucose levels above this range are high (Hi), at termination, urine glucose was quantified via a glucose colorimetric assay kit according to the manufacturer's instructions (Abcam #ab65333).

### Tissue collection and histological analysis

The heart was stopped in diastole with 30 mM (w/v) KCl, flushed with 1% EDTA in normal phosphate-buffered saline (PBS), and cut into pieces; the top section was flash-frozen for future protein and molecular biology studies, and the lower section was drop-fixed in 4% PFA. All the PFA samples were incubated at + 4 °C for 18–24 h, after which the PFA was replaced with PBS. The PFA-fixed cardiac tissues were cryoprotected with 30% sucrose for 24–48 h before being embedded in the OCT compound. Histochemical and immunohistochemical studies were performed on six-μm-thick sections cut with a Thermo Fisher Scientific CryoStar NK50 cryostat unless otherwise stated.

*Histological analyses of mouse hearts*. The specific antibodies and procedures used are listed in Supplementary Table 1. All the immunohistochemical procedures included tissue sections without primary antibodies as a technical internal control and suitable irrelevant IgG as a negative control. Images were acquired via a transmitted light microscope (Olympus BS40), a confocal microscope (Leica TCS-SP8), or an epifluorescence microscope (Zeiss AxioObserver Z1).

Vascular density was measured by counting capillaries and arterioles (Zeiss Observer. Z1 microscope, 20 × objective). The final data are expressed as the number of capillaries and arterioles per mm^2^. Analysis of PDGFRβ + pericytes (PCs) associated with the coronary capillaries and arterioles was carried out on sections stained with anti-PDGFRβ antibodies to identify PCs overnight at + 4 °C (1:50, R&D Systems, #AF1042), with alpha-smooth muscle actin (α-SMA, 1:400, Sigma, #C61198), arteries identified with isolectin-B4 (1:200, Life Technologies, #121,414), endothelial cells identified with α-sarcomeric actin (α-SA, 1:200, IgM isotype, Sigma, #A2172) overnight at + 4 °C. For analysis of PC density, images with capillaries in cross-sections were obtained via a 20 × objective. The density of PCs was expressed as the number of IB4- and α-SMA PDGFRβ + perivascular cells/mm^2^ of the α-SA + myocardial area considered separately PCs around capillaries and arterioles. The cryosections were stained for collagen via the Azan Mallory method (Heidenhain’s adaptation of Mallory’s trichrome stain). The degree of fibrosis was assessed in the perivascular area and quantified as the ratio of collagen to the vessel area. Interstitial fibrosis was also measured in pixels and expressed as a percentage of the tissue area.

A senescence β-galactosidase staining kit (Cell Signaling Technology #9860) was used to identify senescent cells in the frozen cardiac sections (Olympus microscope, × 400 magnification). In addition, senescent cardiomyocytes and interstitial cells were recognized via anti-mouse P16ink4A (1:50, Santa Cruz, #sc-1661) and expressed as the number of positive nuclei per mm^2^ of tissue. Cardiac muscles were stained with α-SA for 2 h at RT, and Alexa Fluor 647-conjugated anti-mouse IgM (1:200, for 1 h, at + 20 °C; Life Technologies, UK) was used as a secondary antibody. Slides were stained with 1:1000 (v/v) DAPI solution in 1 × PBS and mounted with Fluoromount G for imaging. Representative and quantitative images were taken using a Zeiss Observer. A Z1 microscope was set up on a fluorescent field path with a 20 × objective. P16ink4A-positive cells were expressed as the number of positive nuclei per mm^2^ of tissue. Cardiac samples were assessed for apoptosis via a TUNEL assay, according to the manufacturer's instructions (ApopTag® Red In Situ Apoptosis Detection Kit, Millipore #S7165).

For Bpifb4 staining, antigen retrieval was performed on cryosections using citrate buffer (pH = 6; 1x; Sigma‒Aldrich) for 15 min at + 98 °C to unmask the epitopes within the tissue. A blocking solution containing 5% v/v goat serum was used to stop nonspecific binding. Antibody staining was performed using an anti-α-SA antibody (1:200) for two hours at RT to recognize cardiac muscle. Alexa Fluor 647-conjugated anti-mouse IgM (1:200) was used as a secondary antibody. A rabbit anti-Bpifb4 antibody (GeneTex, #GTX51455) was used as the primary antibody at a ratio of 1:100, and Alexa Fluor 568-conjugated anti-rabbit antibody was used at a ratio of 1:200 for Bpifb4 visualization. Nuclei were labelled with DAPI (1:1000), and slides were covered with aqueous Fluoromount G and coverslips for fluorescence imaging. Images were taken via a Leica TCS-SP8 confocal laser scanning microscope attached to a Leica DM I8 inverted epifluorescence microscope and processed via LASX software. Lightning adaptive image restoration was used to optimize the resolution. ImageJ Fiji was used to merge the fluorescent-colored channels and to add scale bars. The staining quantification was performed on the whole heart area and its subregions (inner, middle, and outer cardiac layers), and the results are illustrated separately. All the morphometric analyses were carried out using ImageJ software.

### Mouse SXCL12/SDF-1 assay

The concentration of CXCL-12/SDF-1 in plasma samples was determined using the DuoSet ELISA assay kit according to the manufacturer's instructions (R&D Systems, cat n# DY460, UK).

### Statistical analyses

The data are presented as individual values and means ± standard errors of the means (SEMs) or standard deviations. The D’Agostino‒Pearson and Kolmogorov‒Smirnov normality tests were used to check for a normal distribution when applicable. Echocardiography parameters (baseline and final assessed in the same animal) were evaluated via a mixed-effects model, Two-way ANOVA was used to examine the influence of two different categorical independent variables (treatment and sex) on one continuous dependent variable and then to determine if they were interacting. Post hoc analyses included Tukey’s comparison test. The unpaired t-test was used for all other analyses to compare two groups, except for data that were not normally distributed, for which the Mann‒Whitney U test was used. Significance was assumed when the P value was 0.05 or less. Analyses were performed via GraphPad Prism 10.0 (San Diego, CA, USA).

## Results

### LAV-BPIFB4 protein therapy improves indices of cardiac function in aging mice

**Fig. 1:**
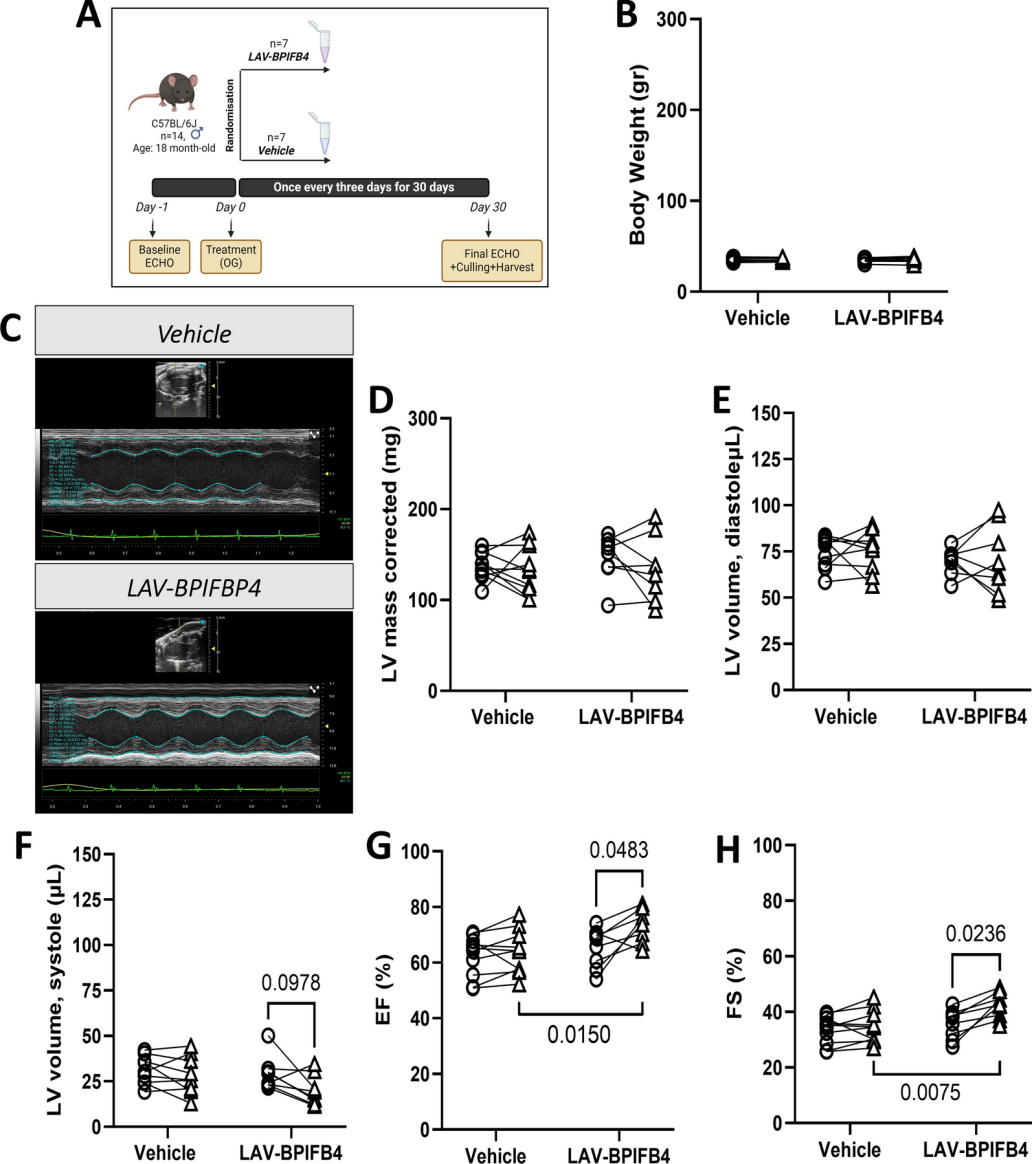
Primary endpoints of the study in older mice. **A** Experimental protocol. **B** Body weight at baseline and the end of the follow-up. **C** Representative echocardiography traces. **D**–**H** Echocardiography data: left ventricular mass (**D**), left ventricular volume at the end of diastole (**E**) and systole (**F**), left ventricular ejection fraction (**G**), and left ventricular fractional shortening (**H**). Each point represents a single mouse, with a line connecting the baseline and final measurements. The mean values and standard deviations are reported in Supplementary Table 2

All the animals that entered the randomization completed the study follow-up, as illustrated in Fig. 1A. No group difference was observed in body weight (Fig. 1B).

An illustrative image of the M-mode echocardiogram is shown in Fig. 1C. At baseline, the two groups presented similar echocardiography parameters. No significant difference in HR or LV mass or volume before and after treatment was detected between the groups, with only the end-diastolic volume showing a decreasing trend in the LAV-treated group (Fig. 1D-F and Supplementary Table 2 reporting the mean values and standard deviations). Notably, the LAV-treated group presented improved indices of LV function, including EF (absolute change from baseline: 8.51 vs 1.60 units in vehicle, *P* < 0.05) and FS (absolute change from baseline: 6.79 vs 1.25 units in vehicle, *P* < 0.05) (Fig. 1G-H and Supplementary Table 2). The analysis of diastolic function was technically challenging, with Doppler images being of good quality in only a fraction of the mice. Hence, although an improvement in the E/A index was observed in the LAV-treated group, this finding should be considered with caution (Supplementary Table 2).

### LAV-BPIFB4 protein therapy induces the expression of endogenous Bpifb4, improves the coronary microvasculature, and reduces fibrosis and senescence in the hearts of aging mice

**Fig. 2:**
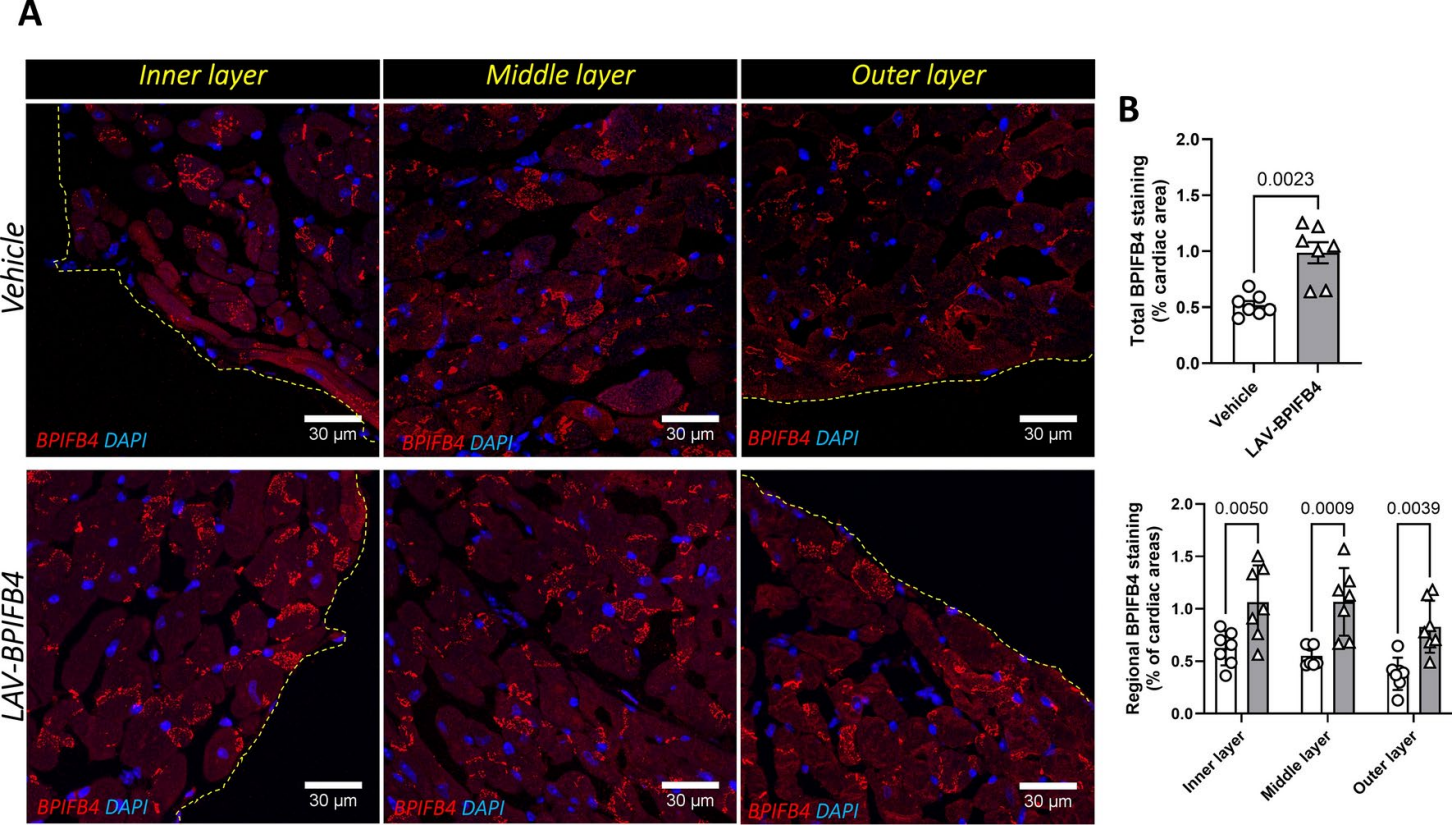
Effect of treatment on the myocardial expression of BPIFB4. **A** Representative images with inserts. **B** Bar graphs with individual values

The heart of LAV-treated mice showed 2.17-fold increased staining for the Bpifb4 protein compared with vehicle-treated mice (Fig. 2).

**Fig. 3:**
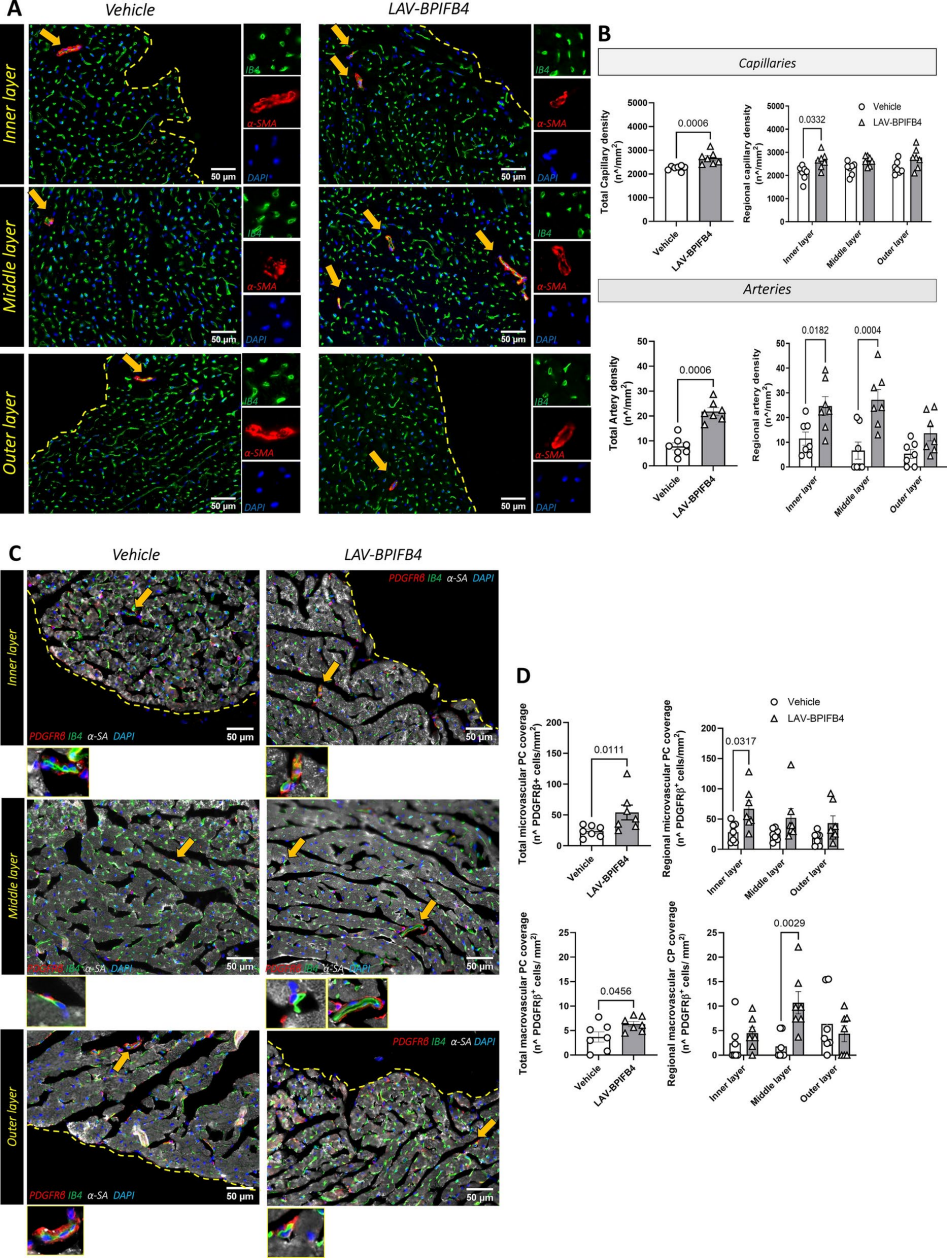

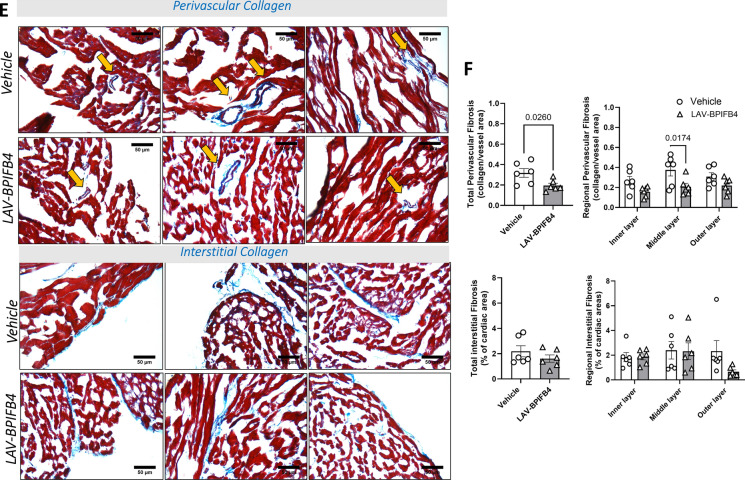
Histological endpoints of the study in older mice. **A** Representative images illustrating the abundance of capillaries and arterioles. The inserts show specific staining. **B** Bar graphs with individual values of capillary and arteriole density in the whole left ventricular wall and single regions of the heart. **C**, **D** Quantification of vessels covered by PDGFRβ PCs. Representative images with inserts (**C**) and bar graphs with individual values for capillary and arteriole coverage in the whole left ventricular wall and single regions of the heart (**D**). **E**, **F** Quantification of fibrosis via Azan-Mallory staining. Representative images (**E**) and bar graphs with individual values of perivascular and interstitial fibrosis (**F**)

The effect of LAV-BPIFB4 protein therapy on microvascular density was analyzed in the whole LV myocardium and then in different layers of the heart, from the epicardium to the endocardium. As previously shown via gene therapy, compared with vehicle treatment, LAV-BPIFB4 treatment increased capillary and arteriole density (Fig. 3A, B). The beneficial effect was evident in the internal layers, whereas no group difference was noted regarding the epicardial vasculature. Moreover, the increase in myocardial microvessel density was associated with greater coverage of cells expressing the mural marker PDGFR-β, which is suggestive of augmented robustness conferred by recruited PCs (Fig. 3C, D). Perivascular fibrosis, another typical feature of the aging heart, is involved in cardiac dysfunction by limiting the oxygen and nutrient supply and washout of metabolites [31, 32]. Although perivascular and interstitial fibrosis often develop together in several cardiovascular diseases, this is not always the case, with implications for therapy [33]. Intriguingly, perivascular fibrosis was reduced by LAV-BPIFB4 protein therapy, with the middle layer of the myocardium showing a significant benefit. In contrast, we did not observe any improvement in the amount of interstitial fibrosis (Fig. 3E, F).

**Fig. 4:**
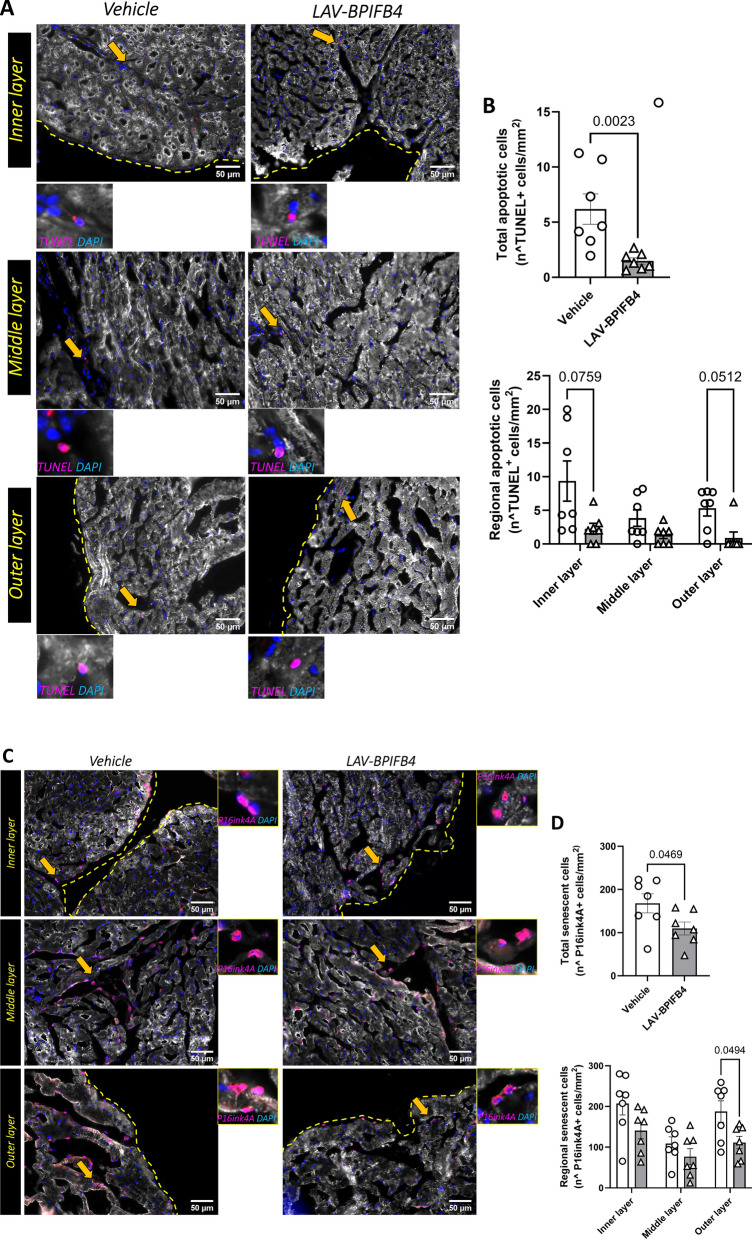
Effect of treatment on markers of viability and senescence. **A**, **B** TUNEL staining for visualization and quantification of apoptotic cells. Representative images with inserts (**A**) and bar graphs with individual values (**B**). **C**, **D** Staining with P16ink4A for visualization and quantification of senescent cells. Representative images with inserts (**C**) and bar graphs with individual values (**D**) are shown. **E**, **F** Staining with SA-β-Gal for visualization and quantification of senescent cells. Representative images with inserts **E** and bar graphs with individual values **F** are shown

Apoptotic and senescent cells, identified by TUNEL and p16Ink4A/β-Gal, respectively, were also reduced in the whole myocardium of LAV-BPIFB4-treated mice. However, regional analysis revealed a less consistent distribution of this phenomenon than microvascular and fibrotic effects (Fig. 4A, F).

### LAV-BPIFB4 protein therapy improves cardiac function in type 2 diabetic mice

**Fig. 5:**
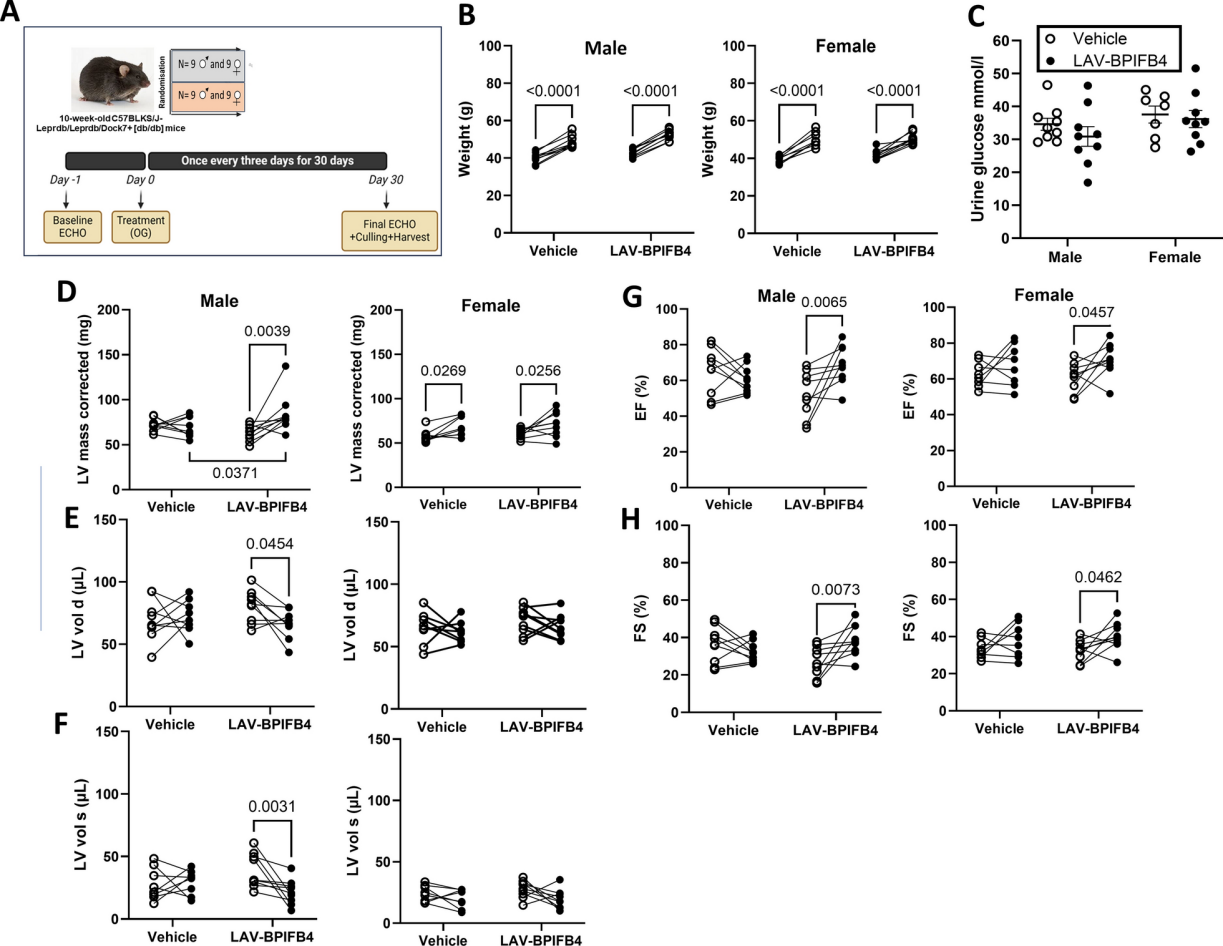
Main endpoints of the study in male and female diabetic mice. **A** Experimental protocol. **B** Body weight at baseline and at the end of the follow-up. **C** Urinary glucose levels. D-H) Echocardiography data: left ventricular mass (**D**), left ventricular volume at the end of diastole (**E**) and systole (**F**), left ventricular ejection fraction (**G**), and left ventricular fractional shortening (**H**). Each point represents a single mouse, with a line connecting the baseline and final measurements. The mean values and standard deviations are reported in Supplementary Table 3

**Fig. 6:**
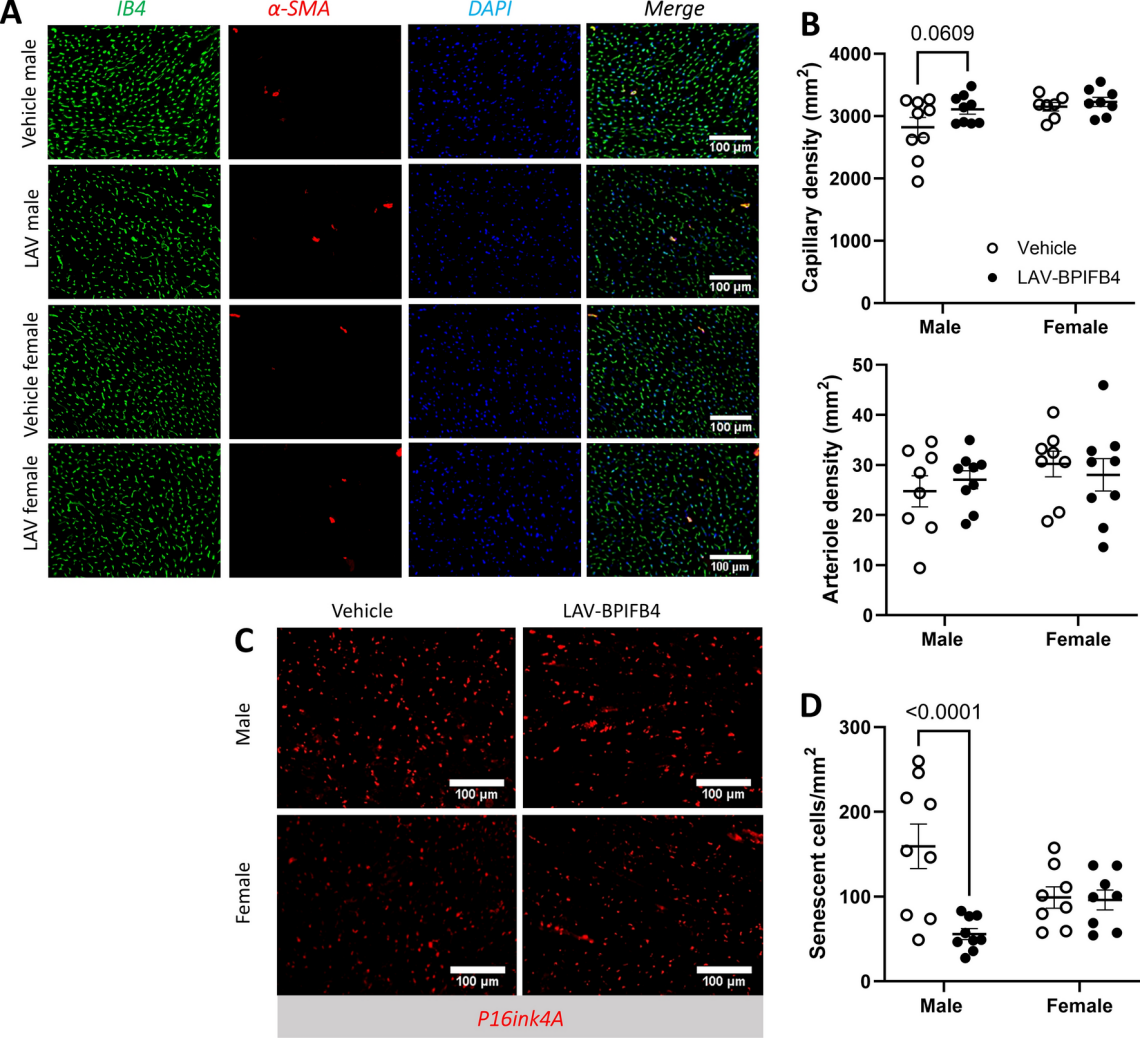
Vascular endpoints of the study in diabetic mice. **A** Representative images illustrating the abundance of capillaries and arterioles. **B** Graphs with individual values of capillary density (**B**) and arteriole density (**B**). **C**, **D** P16inkA + senescent cells (illustrative images (**C**) and graphs (**D**))

Figure 5A illustrates the protocol of this study. There was no group difference in body weight growth during the follow-up (Fig. 5B). Moreover, there was no difference in glycosuria (Fig. 5C) or blood glucose (data not shown). Interestingly, LAV-BPIFB4 protein therapy induced volumetric changes in male mice, increasing the LV mass and decreasing LV end-diastolic and end-systolic volumes. In contrast, we did not observe any difference concerning the females treated with LAV protein or vehicle (Fig. 5D, F and Supplementary Table 3). Moreover, male and female mice presented improved systolic function, as assessed by measurements of EF and FS; however, this effect was more evident in male mice. Consistent with these findings, compared with vehicle-treated male mice, LAV protein-treated male mice tended to have greater capillary density and significantly reduced senescence in the myocardium; however, no therapeutic effect was noted in females (Fig. 6A, B). The treatment did not cause any change in arteriole density in either sex (Fig. 6C, D). To determine a possible effect of SDF1 on the observed sex dimorphism, we measured the circulating levels of this cytokine. We found they were similar in male and female mice treated with vehicle or LAV-BPIFB4 (Supplementary Fig. 1).

## Discussion

Previous work documented the potential of *LAV-BPIFB4* gene therapy for the treatment of cardiovascular diseases and cardiomyopathies [20, 23, 24], More recently, we have gained evidence for the similar therapeutic value of directly using the encoded protein. In vitro conditioning with the LAV-BPIFB4 protein induced macrophages from atherosclerotic patients to acquire an anti-inflammatory phenotype [22]. Furthermore, treatment with the LAV-BPIFB4 protein restored endothelial function, increased eNOS phosphorylation, and rebalanced the levels of anti- and pro-inflammatory cytokines in arteries explanted from atherosclerotic patients [22]. In the present in vivo study, we have newly demonstrated the feasibility and efficacy of a 30-day treatment with the LAV-BPIFB4 protein in older and diabetic mice.

Increased evidence links HFpEF, microvascular disease, and subendocardial ischemia [34]. The PROMIS-HFpEF study reported a 75% prevalence of coronary microvascular disease in HFpEF patients with an impaired coronary flow velocity reserve [35]. In another study on patients hospitalized for HFpEF, coronary microvascular disease was present in 81% of those who did not have obstructive epicardial coronary artery disease [36]. Zooming into the in vivo microvascular effect of LAV-BPIFB4 protein, we assessed the capillary and arteriole density in the LV wall and selected myocardial regions. Interestingly, although endogenous Bpifb4 expression was equally induced in all the heart layers, we found that LAV-BPIFB4 protein therapy promoted significant microvascular enhancements only in the subendocardial region, which is the most vulnerable to ischemia [37, 38]. This regional effect was associated with more PC coverage, less cardiomyocyte apoptosis, and less perivascular fibrosis, replicating the results obtained with *LAV-BPIFB4* gene therapy, which improved coronary artery reserve. [23]

We reported, and other authors have confirmed, a reduction of PC density and coverage in mice starting at 18 months of age [23, 39]. Single-nucleus RNA sequencing analysis of cardiac cells from aging mice further revealed that PCs acquire the expression of various extracellular matrix components and growth factors, such as TGFB2 and PDGFB, suggesting that PC reduction may be due to differentiation in myofibroblasts [39]. This possibility is consistent with the observed perivascular fibrosis, a condition frequently associated with microvascular disease caused by metabolic dysfunction and aging. In contrast, substitutive interstitial fibrosis is more typical in conditions of abundant cardiomyocyte loss, for instance, after a myocardial infarction [40]. Another explanation for PC rarefaction is that these cells are lost by apoptosis. Accordingly, cardiac PCs from elderly patients with HF showed upregulation of senescent markers, an abundance of oxidized lipofuscin, and increased levels of mitochondrial superoxide, a finding that we interpreted as an accumulation of biological ‘garbage’ from oxidative stress [23]. Notably, our in vivo study shows that supplementation with the recombinant LAV-BPIFB4 protein rescued PC defects. In a previous report, the protein conferred human HF-derived PCs with increased capacity to support the formation of networks made of late passage, senescent endothelial cells through increased secretion of pro-angiogenic factors [23]. Novel treatments like LAV-BPIFB4, capable of halting the functional consequences of microvascular dysfunction, perivascular fibrosis, and PC loss in the ageing and diabetic myocardium, could improve the clinical outcome of patients with HFpEF. [40, 41]

Women with diabetes are at greater risk of developing diabetic cardiomyopathy, a condition that often evolves into HFpEF [42]. Our study on obese, type 2 diabetic mice did not reveal significant sex-related differences in cardiac function and structure, except for a higher abundance of senescent cells in male hearts. Intriguingly, we observed a sex dimorphism in the response to LAV-BPIFB4 protein therapy, which was more effective in male mice at both the functional and microvascular levels. Various hypotheses could account for the observed sex-specific variations in treatment efficacy. One possibility is that genetic factors, such as sex-linked genes or disparities in gene expression, influence the therapeutic effects of LAV-BPIFB4. Our previous study in three independent human cohorts did not demonstrate an influence of gender on the association between the *LAV-BPIFB4* gene variant and exceptional longevity [20]. Another possibility is that hormonal disparities, such as varying estrogen or testosterone levels, could influence the response to LAV-BPIFB4, affecting the protein's interaction with cellular receptors or signalling pathways. In this respect, we previously found that SDF-1 upregulation in peripheral blood and myocardium played a pivotal role in the cardioprotective effect of *LAV-BPIFB4* gene therapy in male diabetic mice, as confirmed by abrogation of benefit by administration of an antagonist of the SDF-1 CXCR4 receptor [24]. SDF-1 expression in the rodent heart is modulated by estrogen through the cognate receptor ER-α [43], However, in diabetes, an increased expression and activation of the other estrogen receptor ER-β over the ER-α reportedly contributes to oxidative stress, vascular inflammation, and atherosclerosis [44, 45]. We posited that the shift in estrogen receptors might influence the levels of SDF-1 and consequently diminish LAV-BPIFB4 efficacy in female diabetic mice. Contrary to our prediction, the plasma immunoreactive levels of SDF-1 were similar in male and female diabetic mice and were not modified by the LAV-BPIFB4 therapy. Although not excluding an influence of sex on CXCR4 receptor activity and downstream signalling at the microvascular level, these data do not favor the role of circulating SDF-1 in the therapeutic effect of LAV-BPIFB4. Additional studies are necessary to confirm the intriguing dimorphism and its underpinning mechanisms.

In conclusion, this is the first demonstration that a variant protein associated with human longevity can contrast pivotal risk factors for HFpEF. These findings may open new avenues for the prevention of cardiac deterioration due to advanced age and diabetes.

## Supplementary Information

Additional file1 (DOCX 60 KB)

## Data Availability

No datasets were generated or analysed during the current study.
