# Supplementary material for: Healthy longevity-associated protein improves cardiac function in murine models of cardiomyopathy with preserved ejection fraction
Source: Cardiovasc Diabetol. 2024 Nov 5;23:397. doi: 10.1186/s12933-024-02487-6 (PMC11536962; doi:10.1186/s12933-024-02487-6)
Supplement: Supplementary file 1 — Additional file1 (DOCX 60 KB) [file 12933_2024_2487_MOESM1_ESM.docx]

**Supplementary Table 1. Antibodies and experimental conditions employed for immunohistochemical analyses on mouse sections.**

| Antigen | Company (Catalog N°) | Dilution | Antigen retrieval | Incubation time and temperature | Secondary antibody | | Incubation time | |
| --- | --- | --- | --- | --- | --- | --- | --- | --- |
| α-Sarcomeric Actin | SIGMA (A2172) | 1:200  or 1:100 | Citric buffer (pH6), 98°C, 40’  or none | 1h, 37°C  or 2h, RT  or O/N, 4°C | Cy5 1:200  or Alexa Fluor 647 1:200  or TRITC 1:200 | 1h, 37°C  or 1h, RT | | |
| PDGFRβ | R&D Systems (AF1042) | 1:50 | none | O/N, 4°C | Alexa Fluor 647 1:200 | 1h, RT | | |
| P16ink4A | Santa cruz biotechnology (sc-1661) | 1:50 | none | O/N, 4°C | Alexa Fluor 568 1:200 | 1h, RT | | |
| isolectin gs-Ib_4_-biotinILATED | Life Technologies (121414) | 1:200 | Citric buffer (pH6), 98°C, 30’  or none | O/N, 4°C | Streptavidin- Alexa Fluor 488 1:200 | 1h, RT | | |
| α-smooth muscle actin-Cy3 | Sigma C6198 | 1:400 | Citric buffer (pH6), 98°C, 30’ | O/N, 4°C | N/A | N/A | | |
| BPIFB4 | GeneTex (GTX51455) | 1:100 | Citric buffer (pH6), 98°C, 15’ | O/N, 4°C | Alexa Fluor 568 1:200 | 1h, RT | | |
| Legend: |  | | | | | | |  |
| A488 | Alexa Fluor 488 labeled donkey Antibody OR streptavadin | | | | | | |  |
| A555 | Alexa Fluor 555 labeled donkey Antibody | | | | | | |  |
| A568 | Alexa Fluor 568 labeled donkey OR goat Antibody | | | | | | |  |
| A633 | Alexa Fluor 633 labeled donkey Antibody | | | | | | |  |
| A647 | Alexa Fluor 647 labeled donkey OR goat Antibody | | | | | | |  |
| TRITC | TRITC labeled goat Antibody | | | | | | |  |
| Cy5 | Cy5 labeled donkey Antibody | | | | | | |  |

**Supplementary Table 2: Echocardiography data of the study in older mice**

| **Elderly male** | **Vehicle** | | | **LAV** | | |
| --- | --- | --- | --- | --- | --- | --- |
|  | **basal** | **final** | **fold change** | **basal** | **final** | **fold change** |
| **HR (b/min)** | **449 ± 52** | **454 ± 39** | **1.01± 0.08** | **467 ± 45** | **469 ± 25** | **1.02 ± 0.12** |
| **LV mass (mg)** | **137.5 ± 14.9** | **133.5 ± 26.1** | **0.98 ± 0.23** | **147.1 ± 24.9** | **134.5 ± 35.8** | **0.92 ± 0.21** |
| **LV vol s (μL)** | **31.1 ± 7.9** | **28.4 ± 10.3** | **0.88 ± 0.42** | **29.2 ± 9.3** | **19.9 ± 8.5** | **0.80 ± 0.51** |
| **LV vol d (μL)** | **74.6 ± 8.8** | **74.9 ± 11.3** | **0.91 ± 0.26** | **69.9 ± 7.1** | **70.7 ± 18.4** | **0.71 ± 0.34** |
|  |  |  | **absolute change** |  |  | **absolute change** |
| **LV EF (%)** | **62.3 ± 7.4** | **63.9 ± 7.92** | **1.60 ± 4.99** | **65.2 ± 7.1** | **73.7 ± 5.9*** | **8.51 ± 8.47****^+^** |
| **LV FS (%)** | **33.5 ± 5.1** | **34.7 ± 5.9** | **1.25 ± 3.82** | **35.5 ± 5.8** | **42.3 ± 4.6*** | **6.79 ± 6.45^+^** |
| **E/A** | **1.25 ± 0.08** | **1.51 ± 0.22** | **0.26 ± 0.21** | **1.25 ± 0.06** | **1.82 ± 0.67*** | **0.57 ± 0.32** |

Values are mean and standard deviation

**Supplementary Table 3: Echocardiography data of the study in diabetic mice**

| **Diabetic male** | **Vehicle** | | | **LAV** | | |
| --- | --- | --- | --- | --- | --- | --- |
|  | **basal** | **final** | **fold change** | **basal** | **final** | **fold change** |
| **HR (b/min)** | **422 ± 37** | **395 ± 42** | **0.94 ± 0.12** | **423 ± 25** | **440 ± 20** | **1.04 ± 0.06** |
| **LV mass (mg)** | **72.3 ± 6.9** | **69.1 ± 11.3** | **0.89 ± 0.19** | **62.9 ± 8.9** | **84.8 ± 21.5**** | **1.37 ± 0.37^++^** |
| **LV vol s (μL)** | **25.6 ± 13.9** | **28.9 ± 9.3** | **1.54 ± 1.02** | **39.0 ± 13.7** | **21.5 ± 10.1*** | **0.62 ± 0.31^++^** |
| **LV vol d (μL)** | **69.5 ± 16.6** | **72.3 ± 12.7** | **1.11 ± 0.12** | **80.6 ± 13.0** | **66.1 ± 11.6*** | **0.84 ± 0.21** |
|  |  |  | **absolute change** |  |  | **absolute change** |
| **LV EF (%)** | **65.1 ± 13.3** | **60.7 ± 7.8** | **-4.34 ± 13.40** | **52.2 ± 12.9** | **68.8 ± 10.8**** | **16.6 ± 18.1^++^** |
| **LV FS (%)** | **36.1 ± 9.4** | **32.3 ± 5.6** | **-3.74 ± 10.08** | **27.0 ± 8.2** | **38.7 ± 8.6**** | **11.7 ± 12.64^+^** |
| **E/A** | **0.99 ± 0.47** | **0.82 ± 0.24** | **-0.18 ± 0.50** | **0.86 ± 0.23** | **0.94 ± 0.26** | **0.07 ± 0.41** |

| **Diabetic female** | **Vehicle** | | | **LAV** | | |
| --- | --- | --- | --- | --- | --- | --- |
|  | **basal** | **final** | **fold change** | **basal** | **final** | **fold change** |
| **HR (b/min)** | **424 ± 42** | **424 ± 32** | **1.00 ± 0.06** | **420 ± 32** | **424 ± 30** | **1.01 ± 0.06** |
| **LV mass (mg)** | **57.9 ± 7.2** | **68.8 ± 10.9*** | **1.19 ± 0.20** | **61.2 ± 5.5** | **71.5 ± 14.2*** | **1.17 ± 0.23** |
| **LV vol s (μL)** | **24.1 ± 6.7** | **20.1 ± 7.8** | **0.88 ± 0.42** | **27.7 ± 7,1** | **19.6 ± 7.8** | **0.80 ± 0.51** |
| **LV vol d (μL)** | **64.9 ± 13.2** | **61.7 ± 12.6** | **0.99 ± 0.28** | **70.8 ± 10.6** | **66.1 ± 9.9** | **0.95 ± 0.20** |
|  |  |  | **absolute change** |  |  | **absolute change** |
| **LV EF (%)** | **62.7 ± 7.3** | **67.8 ± 11.6** | **5.05 ± 13.18** | **61.0 ± 8.3** | **70.8 ± 9.2*** | **9.74 ± 13.62** |
| **LV FS (%)** | **33.6 ± 5.4** | **37.9 ± 9.3** | **4.33 ± 10.73** | **32.5 ± 5.8** | **40.1 ± 7.4*** | **7.59 ± 10.28** |
| **E/A** | **0.83 ± 0.22** | **0.85 ± 0.16** | **-0.12 ± 0.35** | **0.96 ± 0.28** | **0.91 ± 0.30** | **-0.13 ± 0.50** |

Values are mean and standard deviation

**Supplementary Figure 1: Circulating levels of immunoreactive SDF-1.**
